# P-1905. Quality of Life 6 Months After SARS-CoV-2 Infection in Ambulatory and Hospitalized Patients from Lima, Peru: FUNCTION Cohort Study

**DOI:** 10.1093/ofid/ofae631.2066

**Published:** 2025-01-29

**Authors:** Carolina Coombes, Rodrigo Antonio A Cachay Figueroa, Takashi Watanabe, Isabel Ballena, Fernando Mejia, Felix Medina, Oscar Gayoso, Carlos Seas, Larissa Otero, Eduardo Gotuzzo

**Affiliations:** Instituto de Medicina Tropical Alexander von Humboldt - UPCH, LIMA, Lima, Peru; Instituto de Medicina Tropical Alexander von Humboldt - UPCH, LIMA, Lima, Peru; Instituto de Medicina Tropical Alexander von Humboldt - UPCH, LIMA, Lima, Peru; Radiology Department, Clinica Medica Cayetano Heredia, Lima, Lima, Peru; Instituto de Medicina Tropical Alexander von Humboldt - UPCH, LIMA, Lima, Peru; Hospital Cayetano Heredia, Lima, Lima, Peru; Pulmonology Department, Hospital Cayetano Heredia, Lima, Lima, Peru; Instituto de Medicina Tropical Alexander von Humboldt - UPCH, LIMA, Lima, Peru; Instituto de Medicina Tropical Alexander von Humboldt - UPCH, LIMA, Lima, Peru; Instituto de Medicina Tropical Alexander von Humboldt, Universidad Peruana Cayetano Heredia, Lima, Lima, Peru

## Abstract

**Background:**

COVID-19 survivors overcome long-lasting symptoms that affect their quality of life (QoL). We assessed the impact on QoL and the factors associated with the persistence and reversibility of poor QoL 6 months after acute COVID 19.

**Figure:**
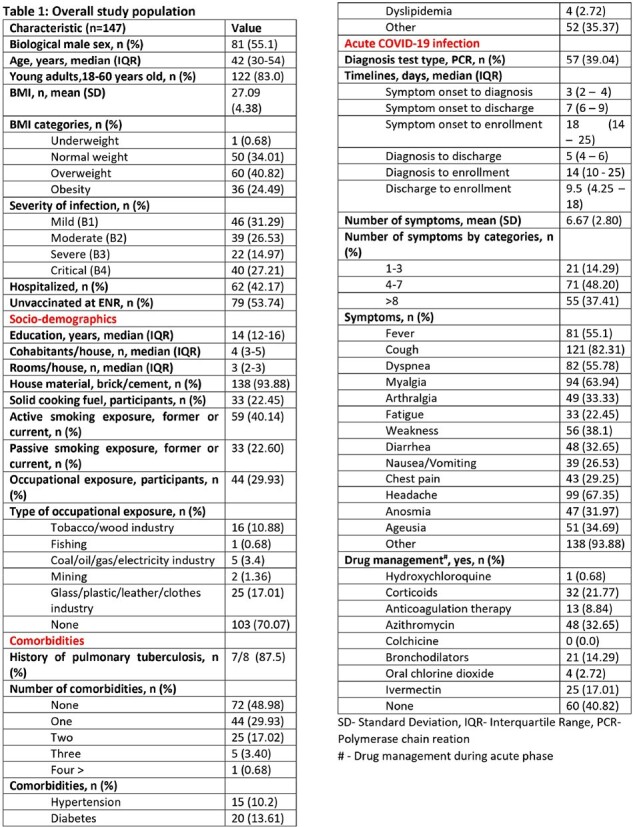

**Methods:**

We enrolled symptomatic patients who tested positive for SARS-COV-2 with an antigenic or RT-PCR test between August 2021 and October 2022. We classified subjects according to disease severity: B1 (mild: outpatient), B2 (moderate: outpatient with pneumonia), B3 (severe: inpatient with oxygen supply) and B4 (critical: inpatient admitted to intensive care unit). Measurements were taken at baseline (BL), month 1 (M1), 3 (M3), and 6 (M6) after acute infection. We evaluated QoL with the RAND-36 questionnaire which provides a score for the Physical Component Score (PCS) and the Mental Component Score (MCS). The PCS comprises scales for physical functioning, role limitations due to physical health, pain and general health. MCS includes scales of role limitations due to mental health, energy/fatigue, emotional wellbeing, and social functioning. PCS and MCS scores were standardized on a 0-100 scale and poor QoL was considered < 40 in each scale.

**Figure:**
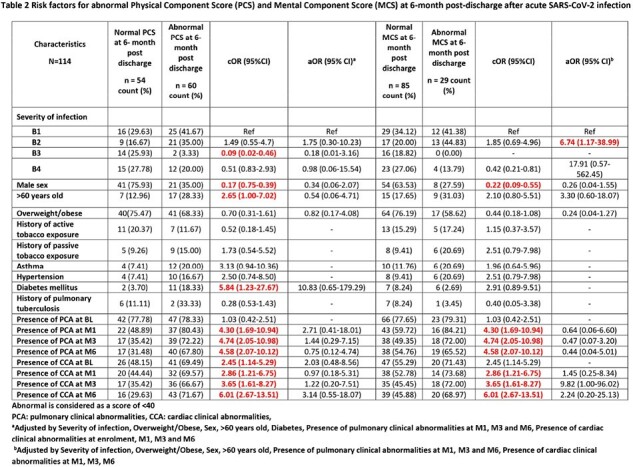

**Results:**

We enrolled 147 participants: 46 in B1 (31%), 39 in B2 (27%), 22 in B3 (15%), and 40 in B4 (27%). We lost to follow-up 33 participants (22.5%) from BL to M6. Significant improvements in PCS were observed between BL and M6 within B3 [35.77 (95%CI 29.7-41.8) vs 51.63 (95%CI 47.1-56.2), p=0.0002] and B4 [28.8 (95%CI 24.5-33.2) vs 43.3 (95%CI 39.4-47.2), p< 0.0001]. In the MCS, significant improvement occurred within B3 between BL and M6 [44.0 (95%CI 36.9-51.0) vs 54.4 (95%CI 49.89-58.86) p=0.0229]. Low MCS and PCS at M6 after COVID-19 was associated with both pulmonary and cardiac clinical abnormalities at M1, M3 and M6. Additionally, only low PCS at M6 was associated with diabetes, age older than 60 years and cardiac clinical abnormalities at BL. Patients who reversed their poor QoL at M6 to a normal value were 122 (83%) for MCS and 93 (63.27%) for PCS. Factors associated with increased reversibility in both scales were severity group B3 and male sex.

**Figure:**
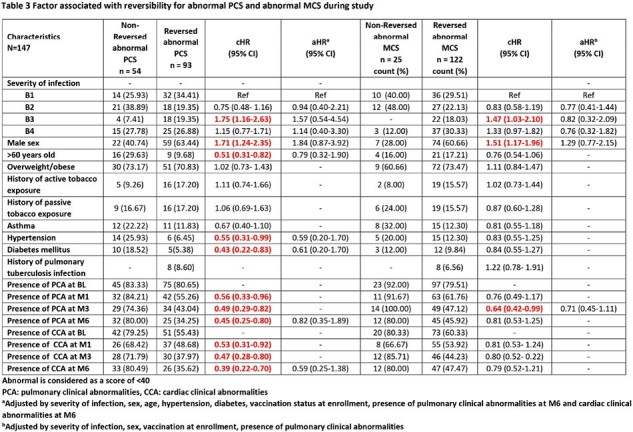

**Conclusion:**

Our findings, the first cohort study on post COVID-19 from Peru, underscore the need for ongoing physical and mental patient monitoring beyond the acute phase of COVID-19.

**Figure:**
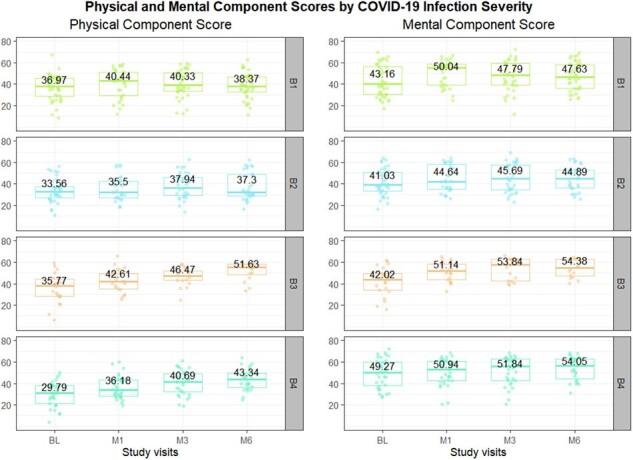

**Disclosures:**

All Authors: No reported disclosures

